# When Rapid Response Becomes Catastrophic: Tumor Lysis Syndrome Following Cisplatin-Etoposide Therapy in Extensive-Stage Small Cell Lung Cancer

**DOI:** 10.7759/cureus.109632

**Published:** 2026-05-25

**Authors:** Nickolle A Cruz Figueroa, Gerardo Martinez Arroyo, Gonzalo Martinez Ruiz, Jean C Rivera, Paloma Delgado, Mileidy Hernandez, Jose Torres Cintron

**Affiliations:** 1 Internal Medicine, Universidad Central del Caribe, Bayamón, PRI

**Keywords:** cisplatin, etoposide, neuroendocrine tumor, oncology emergency, small cell lung cancer, tumor lysis syndrome

## Abstract

Tumor lysis syndrome (TLS) is an oncologic emergency characterized by severe metabolic derangements resulting from rapid tumor cell breakdown. We present the case of a 66-year-old man with extensive-stage small cell lung cancer (SCLC) involving the mediastinum, pleura, and left upper lobe, who developed TLS shortly after initiation of cisplatin-etoposide chemotherapy. Laboratory evaluation demonstrated hyperuricemia, hyperkalemia, hyperphosphatemia, syndrome of inappropriate antidiuretic hormone secretion (SIADH)-related hyponatremia, and acute kidney injury, consistent with TLS. Imaging revealed extensive intrathoracic disease with a large left pleural effusion and mediastinal involvement. The patient required aggressive electrolyte management and close monitoring following chemotherapy initiation. This case emphasizes the importance of maintaining clinical suspicion for TLS in patients with bulky SCLC receiving cytotoxic therapy and highlights the need for early recognition and prompt intervention.

## Introduction

Tumor lysis syndrome (TLS) is an oncologic emergency caused by the rapid destruction of malignant cells, leading to the release of intracellular contents into the bloodstream. The resulting metabolic abnormalities, including hyperuricemia, hyperkalemia, hyperphosphatemia, and hypocalcemia, may lead to cardiac arrhythmias, seizures, acute kidney injury, and death if not recognized promptly. TLS is most commonly associated with hematologic malignancies, such as acute leukemias and high-grade lymphomas, due to their rapid proliferation and high sensitivity to cytotoxic therapy [1,2].

Small cell lung cancer (SCLC) is an aggressive neuroendocrine malignancy characterized by rapid cellular turnover, early metastatic spread, and marked chemosensitivity, all of which may predispose patients to TLS following treatment initiation [3,4]. Although less frequently reported in solid tumors, TLS has been described in patients with SCLC, particularly in cases with extensive disease burden and rapid response to chemotherapy [5]. Because TLS is less expected in solid tumors, recognition may be delayed, increasing the risk of severe complications and mortality [2].

We present a case of TLS occurring shortly after initiation of cisplatin-etoposide chemotherapy in a patient with extensive-stage SCLC involving the mediastinum, pleura, and left upper lobe. While TLS is predominantly observed in hematologic cancers, its occurrence in solid tumors such as SCLC is uncommon. Literature reviews have documented only a relatively small number of reported cases, highlighting the rarity of this complication in SCLC despite its rapid proliferation and high responsiveness to chemotherapy [6]. This case highlights the importance of early recognition and close metabolic monitoring in high-risk patients with bulky SCLC undergoing systemic therapy.

## Case presentation

A 66-year-old man with a recent diagnosis of extensive-stage SCLC with mediastinal and hepatic involvement presented to the ED with progressive generalized weakness, decreased oral intake, nausea, and worsening dyspnea approximately 48 hours after initiation of chemotherapy with cisplatin and etoposide. The patient had recently undergone diagnostic bronchoscopy with biopsy confirming high-grade neuroendocrine carcinoma consistent with SCLC. Immunohistochemical staining was positive for synaptophysin and chromogranin A, with a Ki-67 proliferative index estimated at approximately 60%, supporting highly aggressive tumor biology.

On presentation, vital signs demonstrated a blood pressure of 98/61 mmHg, heart rate of 118 beats/minute, respiratory rate of 26 breaths/minute, temperature of 36.8°C, and oxygen saturation of 91% on room air. Physical examination revealed an ill-appearing man with dry mucous membranes, tachycardia, diffuse weakness, and mild respiratory distress. Pulmonary examination demonstrated decreased breath sounds bilaterally without wheezing. The patient was intermittently confused but remained arousable and oriented to person and place.

**Figure 1:**
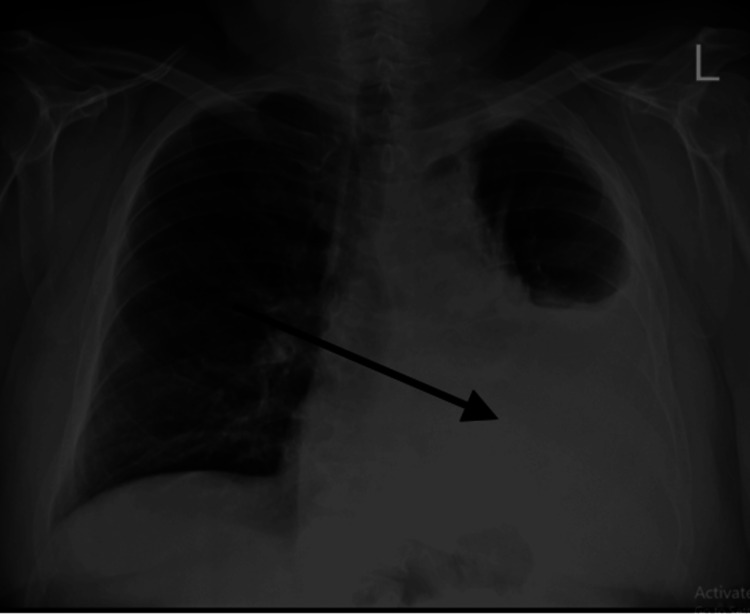
Chest X-ray showing an extensive lung mass compressing the left lung.

**Figure 2:**
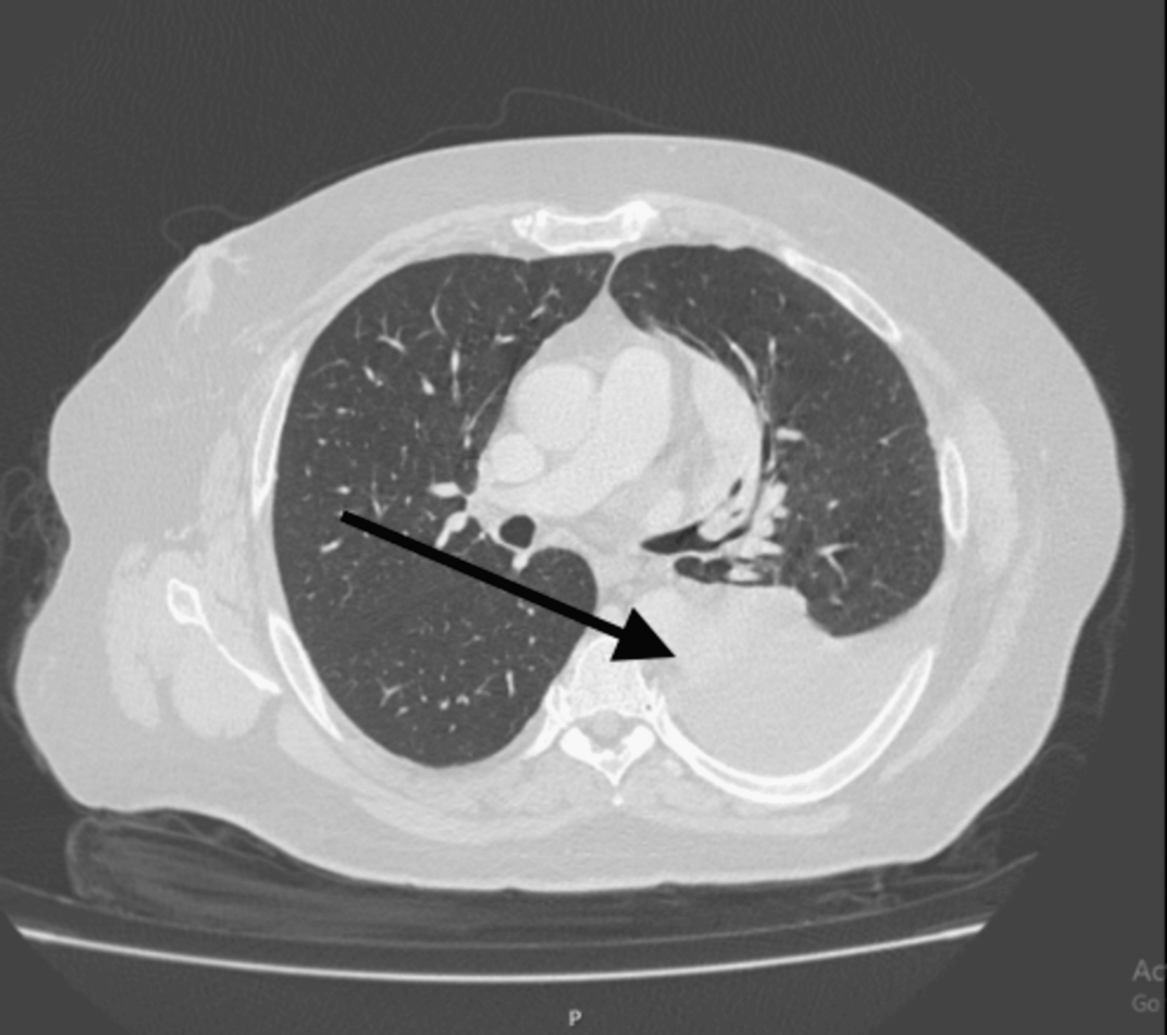
CT scan showing a lung mass with mediastinal involvement and extensive metastatic disease.

Initial laboratory evaluation demonstrated severe metabolic derangements concerning for TLS, including hyperkalemia, hyperuricemia, hyperphosphatemia, elevated lactate dehydrogenase (LDH), and acute kidney injury (Table 1). ECG demonstrated peaked T waves consistent with hyperkalemia. Repeat laboratory studies showed rapid worsening of electrolyte abnormalities and renal function despite initial fluid resuscitation. The chest radiograph demonstrated a large left-sided pleural effusion with associated compressive atelectatic changes and mediastinal obscuration, findings consistent with advanced intrathoracic malignancy burden (Figure 1). CT imaging revealed extensive metastatic disease with high tumor burden, placing the patient at elevated risk for chemotherapy-associated TLS (Figure 2).

**Table 1:** Laboratory findings on presentation.

Laboratory test	Patient value	Reference range
Potassium	6.2 mmol/L	3.5-5.0 mmol/L
Phosphorus	6.8 mg/dL	2.5-4.5 mg/dL
Uric acid	11.2 mg/dL	3.5-7.2 mg/dL
Creatinine	3.1 mg/dL	0.7-1.3 mg/dL
Blood urea nitrogen	48 mg/dL	7-20 mg/dL
Lactate dehydrogenase (LDH)	1,540 U/L	140-280 U/L
Calcium	7.1 mg/dL	8.5-10.5 mg/dL
WBC count	14.8 × 10³/µL	4.0-11.0 × 10³/µL
Hemoglobin	11.4 g/dL	13.5-17.5 g/dL
Platelet count	212 × 10³/µL	150-400 × 10³/µL

Shortly after admission, a rapid response was initiated after the patient developed worsening tachypnea, progressive lethargy, diaphoresis, and cardiac telemetry abnormalities concerning for severe electrolyte-mediated arrhythmogenic risk. The patient required emergent management with IV calcium gluconate, insulin with dextrose, sodium bicarbonate, albuterol nebulization, aggressive IV hydration, and potassium-binding therapy. Nephrology and intensive care specialists were urgently consulted.

Given the patient’s significant tumor burden, markedly elevated LDH, extensive-stage disease with hepatic metastases, and the known chemosensitivity of SCLC, he was recognized as being at elevated risk for TLS before initiation of chemotherapy. IV hydration and close laboratory monitoring were implemented at the time of treatment initiation. However, prophylactic rasburicase was not administered before chemotherapy, as TLS was initially considered less likely given the solid tumor histology. Following the rapid development of metabolic derangements consistent with TLS, aggressive supportive management was initiated promptly, including correction of electrolyte abnormalities and uric acid-lowering therapy. Broad metabolic monitoring was continued with serial electrolyte measurements every four to six hours. Due to worsening renal dysfunction and persistent electrolyte abnormalities, the patient was transferred to the intensive care unit for close hemodynamic and metabolic monitoring, with consideration for renal replacement therapy if clinical deterioration persisted.

The patient gradually demonstrated improvement in potassium and uric acid levels following aggressive medical management. Renal function stabilized over the subsequent days without the need for emergent dialysis. His mental and respiratory status improved with correction of the metabolic abnormalities. The constellation of hyperuricemia, hyperkalemia, hyperphosphatemia, acute kidney injury, elevated LDH, and temporal association with chemotherapy initiation fulfilled clinical and laboratory criteria for TLS according to the Cairo-Bishop classification.

## Discussion

TLS is classically associated with hematologic malignancies such as acute leukemias and high-grade lymphomas; however, increasing evidence demonstrates that TLS can also occur in solid tumors, particularly in malignancies with high proliferative activity, extensive tumor burden, and marked chemotherapy responsiveness [4]. Small cell lung carcinoma represents a particularly aggressive neuroendocrine malignancy characterized by rapid doubling time and high sensitivity to cytotoxic chemotherapy, placing certain patients at increased risk despite TLS remaining relatively uncommon in solid tumors.

Risk factors associated with worse outcomes include elevated baseline LDH, hepatic metastases, preexisting renal dysfunction, dehydration, and bulky disease burden. Although TLS is more commonly associated with hematologic malignancies, it is increasingly recognized in highly proliferative solid tumors such as SCLC. In SCLC, TLS remains uncommon but is well described, particularly in patients with bulky disease, extensive metastatic burden, elevated LDH levels, and rapid response to cytotoxic therapy. Delayed recognition may still occur because the risk of TLS is often underappreciated in solid tumors compared with hematologic cancers, contributing to substantial morbidity and mortality.

The Cairo-Bishop classification remains the most widely used diagnostic framework for TLS and incorporates both laboratory and clinical criteria. In this patient, the presence of hyperuricemia, hyperkalemia, hyperphosphatemia, hypocalcemia, and acute kidney injury shortly after chemotherapy strongly supported the diagnosis of clinical TLS [7]. Cisplatin-etoposide therapy has been reported as a precipitating factor for TLS in extensive-stage SCLC due to the tumor’s rapid proliferative rate and marked chemosensitivity [8]. Early recognition and prompt initiation of aggressive hydration, electrolyte correction, and uric acid-lowering therapy are critical in preventing fatal complications such as cardiac arrhythmias, seizures, and irreversible renal failure.

This case further emphasizes the importance of multidisciplinary collaboration among oncology, nephrology, critical care, and emergency medicine teams when managing rapidly evolving oncologic emergencies. Recognition of TLS risk in aggressive solid tumors may improve outcomes through early prophylaxis, close metabolic surveillance, and timely escalation of care.

## Conclusions

TLS is an uncommon but established complication of extensive-stage SCLC, particularly in patients with bulky tumor burden, elevated LDH levels, hepatic metastases, and rapid chemosensitivity. This case reinforces the importance of recognizing SCLC as a solid tumor subtype in which TLS risk is clinically relevant in selected high-risk patients. Early risk stratification, prophylaxis when appropriate, close laboratory monitoring after chemotherapy initiation, and prompt multidisciplinary management are essential for reducing TLS-related morbidity and mortality.
